# Inkjet-printed fully customizable and low-cost electrodes matrix for gesture recognition

**DOI:** 10.1038/s41598-021-94526-5

**Published:** 2021-07-22

**Authors:** Giulio Rosati, Giulia Cisotto, Daniele Sili, Luca Compagnucci, Chiara De Giorgi, Enea Francesco Pavone, Alessandro Paccagnella, Viviana Betti

**Affiliations:** 1 Department of Information Engineering, University of Padova, via G. Gradenigo 6b, 35131 Padova, Italy; 2 Department of Psychology, University of Rome “La Sapienza”, Piazzale Aldo Moro 5, 00185 Rome, Italy; 3 BrainTrends srl, Fondazione Santa Lucia, 306/354, 00179 Rome, Italy; 4 IRCCS Fondazione Santa Lucia, Via Ardeatina, 306/354, 00179 Rome, Italy; 5 NCNP, National Centre of Neurology and Psychiatry, Tokyo, Japan; 6 CNIT, the National, Inter-University Consortium for Telecommunications, Rome, Italy

**Keywords:** Motor control, Biomedical engineering, Electrical and electronic engineering, Electronic properties and materials, Nanoparticles

## Abstract

The use of surface electromyography (sEMG) is rapidly spreading, from robotic prostheses and muscle computer interfaces to rehabilitation devices controlled by residual muscular activities. In this context, sEMG-based gesture recognition plays an enabling role in controlling prosthetics and devices in real-life settings. Our work aimed at developing a low-cost, print-and-play platform to acquire and analyse sEMG signals that can be arranged in a fully customized way, depending on the application and the users’ needs. We produced 8-channel sEMG matrices to measure the muscular activity of the forearm using innovative nanoparticle-based inks to print the sensors embedded into each matrix using a commercial inkjet printer. Then, we acquired the multi-channel sEMG data from 12 participants while repeatedly performing twelve standard finger movements (six extensions and six flexions). Our results showed that inkjet printing-based sEMG signals ensured significant similarity values across repetitions in every participant, a large enough difference between movements (dissimilarity index above 0.2), and an overall classification accuracy of 93–95% for flexion and extension, respectively.

## Introduction

Surface electromyography (sEMG) represents a classical and non-invasive approach to investigate the integrity and the functionality of muscular activity. sEMG is typically acquired using two electrodes placed over the skin, measuring the voltage difference between the two locations above the target muscle. sEMG can acquire muscular activity from any muscle during motor tasks, i.e., grasping an object, limb movements, and even gait. Its usage is rapidly evolving, especially for bioengineering applications, including robotic prostheses, interfaces for muscle computer interface (MCI), and rehabilitation devices controlled by residual muscular activities. Combined with the advantages offered by printed sensors and electronics, i.e., the ultra-short design-to-prototype time and the customization, sEMG is now rapidly evolving from a laboratory setting to wearable applications^1^. Silver nanoparticle (AgNP) based inks are the most abundant in the market and easy to produce, and for these reasons, they have been extensively used for rapid prototyping of sEMG electrodes^2–7^. Many other nanomaterials emerged in the last years with very promising electrical properties, for instance, graphene, silver nanowires, and carbon nanotubes. However, when it comes to fabrication, they all show significant limitations like lack of batch-to-batch reproducibility, limited conductivity, instability, and typically high costs. Commercial AgNPs inks conversely are very stable and reproducible, guaranteeing higher performances. Although this technology has opened new possibilities for neuroscience to measure sEMG during real-life behaviour^8^, its employment is still limited due to complicated fabrication procedures, expensive equipment materials, and, lastly, strong preparation and expertise^9^. Excellent results have recently been obtained with this approach, for example, using sEMG matrices for human–machine interface in patients with loss of voice; these applications are of interest for voice synthesis and virtual interaction^10^. However, as demonstrated by that one and several other works two main points has to be considered for human machine interface. First, the virtual/electronic/mechanic actuation of the classification results is not the bottleneck of the human–machine interface (as we detail in the outlook)^11–13^. Second, the transduction of the signal is still critical, requiring complicated and expensive fabrication methods, often based on clean-room equipment and long protocols, and still not directly implementable/customizable by the clinician.

Here we propose a paradigm shift in the prototyping and production of sEMG printable sensors introducing a “print and play” fabrication technology. With this approach, any laboratory can exploit the ultra-rapid and low-cost fabrication of customizable systems for electrophysiological measurements, even in the absence of specific expertise. The idea is to provide clinicians with a fabrication platform constituted by a set of easy-to-use tools. This platform will allow users to focus on the applications and be independent of the fabrication of the sensors. With this aim, we are able to optimize every step of the process to keep the costs low and all the materials commercially available (accessible and guaranteed on the supplier website, without any preparation needed after the purchase). In a preliminary study^14^, we investigated the reliability of inkjet-printed electrodes for sEMG compared with Ag/AgCl standard electrodes. We analyzed the fatigue induced by the repeated lifting of loads with variable weights by measuring the individual maximal voluntary isometric contraction (MVIC) value in six participants. In line with sEMG clinical guidelines, MVIC, i.e., the maximal muscular activation exerted by a subject in a specific motor task, was measured from each individual and used to normalize his/her sEMG activity to compare fatigue across different subjects fairly. Results showed that inkjet-printed electrodes were reliable for recording sEMG activity with accuracy and signal quality comparable with standard electrodes.

Based on these findings, in this study, we used the same printed sensors while subjects performed several standard gestures, i.e., extension and flexion of the fingers, as in a previous work^15^ (detailed in the Methods section, where Fig. 6 displays the specific gestures included in the study and univocal codes associated to each of them). In order to test the quality of the EMG signal recorded by inkjet-printed electrodes, we investigated the inter-trial and inter-subject variability of the EMG signals, and we used machine learning to classify gestures. Previous studies have already shown that hand gestures of a specific subject can be effectively recognized using an automatic classification algorithm based on sEMG recordings and that specific patterns of EMG activity are reproducible across trials of the same gesture^16,17^. Here, we investigated the reliability of the sEMG signals acquired using the inkjet-printed electrodes by computing (i) a similarity index across signals of different repetitions of the same gesture in every subject, (ii) a dissimilarity metric between different gestures in every subject, and (iii) the classification performance of three different classifiers that were trained to recognize gesture-specific sEMG patterns across subjects.

For both extension and flexion tasks, we found high similarity values across repetitions for most of the gestures and the majority of our subjects; moreover, the dissimilarity between gestures was large enough to distinguish them. Finally, these findings were confirmed by very high classification accuracies (93% for flexion, 95% for extension), as well as high values of precision (85.4–100% for DA classifier in flexion, 86.8–100% for DA in extension) and recall (88.9–100% for DA in flexion, 84.8–100% for DA in extension) for all gestures. Therefore, we proved the robustness of our inkjet-based sEMG measurements, thus paving the road to a new, more flexible way to acquire sEMG for movement control and gesture recognition applications.

## Results and discussion

This section will report the qualitative observations on the quality of the inkjet-printed sEMG signals, the outcomes from the inter-trials and inter-subjects variability, and the most relevant classification performance.

### Visual inspection of the dataset

First, we evaluated the quality of raw data by visual inspection of the bubble plots, i.e., a brand-new representation showing for each participant and movement the average and standard deviation of the root mean square (RMS) values across repetitions. RMS was chosen among the available features since it is often used as a signal activity index^18^.

**Figure 1:**
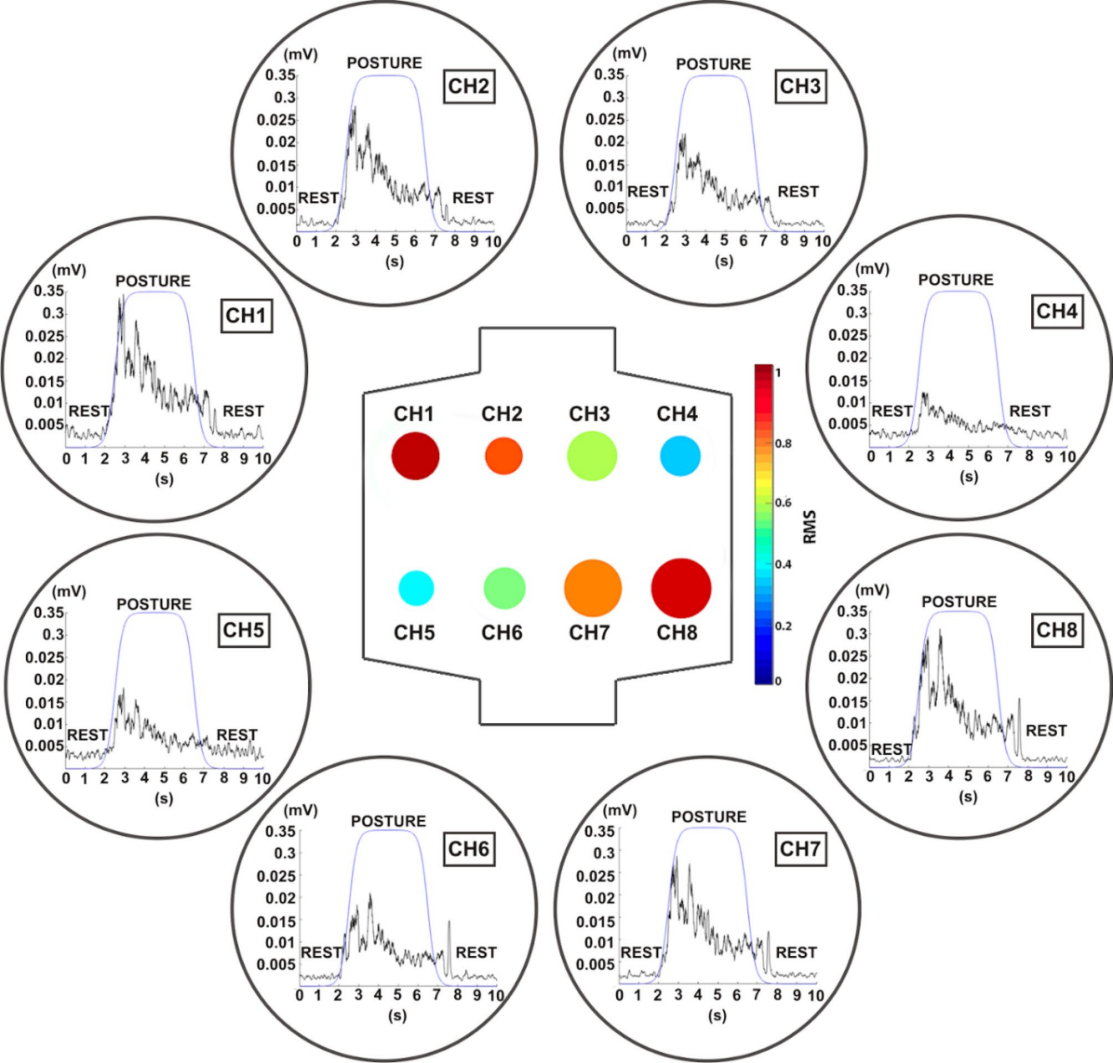
Representative bubble plot showing the signal quality during the flexion movement of the middle finger in a single participant. Each bubble shows the average normalized RMS value across repetitions of the same movement (color code) and its standard deviation (diameter). The corresponding average sEMG signal across trials is shown for each unipolar channel. Figure created with MatLab 2020a and Microsoft PowerPoint 2016.

Figure 1 shows a representative bubble plot for flexion movement of the middle finger (F2) of a single participant; Figure S1 and S2 resumes all the bubble plots of each flexion and extension exercise performed by the same subject.

We compared the signal amplitude differences during the task and rest phase for each repetition of the movements for each participant. Amplitude differences of at least one order of magnitude were found (not reported here for space constraints).

We observed a shift toward low frequency in the rest condition completely lost after 100 Hz, mainly due to membrane potential effects, DC offset, sensor movements, and temperature. A more powerful PSD was noticed between 50 and 150 Hz during the task. In addition to spectral analysis, we assessed the average power in the signal estimating the integral of the PSD over the frequency band 15–250 Hz, evaluating power values along with the repetitions, and comparing them with the rest phases. We detected consistent amplitude differences between the task and the rest phase from spectral analysis and average power.

### Intra-subject and inter-subject variability

**Figure 2:**
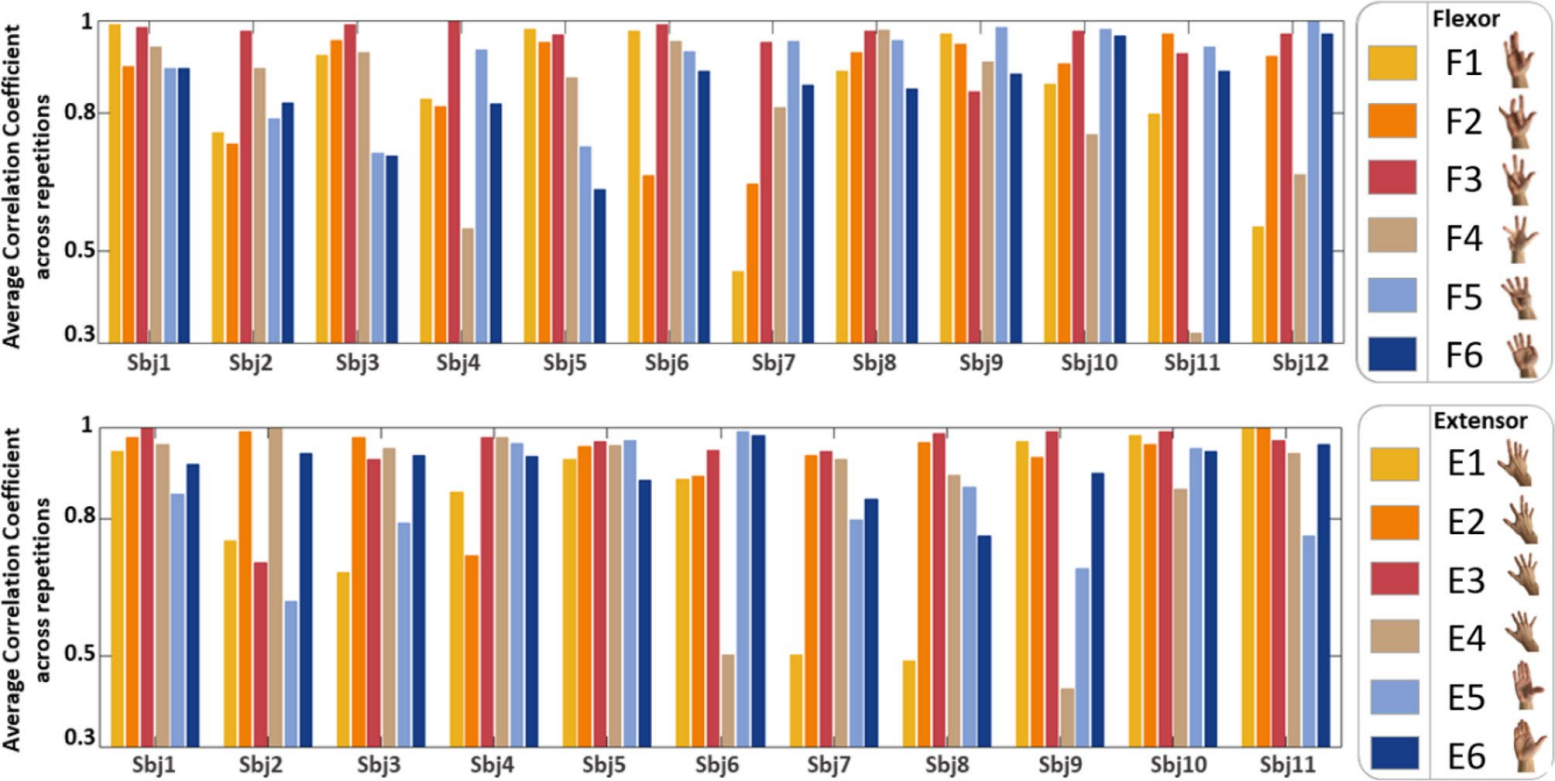
Intra-subject similarity analysis for the flexion (F) and extension (E) tasks. The similarity coefficient (averaged across repetitions) is shown for all the gestures (color code), and subjects, for the flexion (upper panel) and extension (lower panel) sessions, separately. Note that the y-axis ranges from 0.3 to 1. Figure created with Matlab 2020a.

Figure 2 shows the results from the intra-subject similarity analysis. The similarity coefficient (averaged across repetitions) is shown for all the gestures and subjects, for the flexion (F – Fig. 6) and extension (E – Fig. 6) sessions, separately. The analysis of the intra-subject variability showed, regardless of the task, a coefficient of similarity across repetitions higher than 0.45 for the flexion tasks and 0.54 for the extension tasks. Specifically, for the flexors, the subjects 1, 8, 9, and 10 highlight, for all tasks, better repeatability with a coefficient of similarity higher than 0.8. For the extensors, subjects 1, 5, 10, and 11 verify the same condition. The matrices with the similarity coefficient between each pair of repetitions and two representative subjects are shown in Figure S3.

The statistical analysis carried out confirmed the significance of the similarity across the repetitions with a p-value < 0.05, for each subject and each task, except for the subject 11 task 4 (little finger flexion) for the flexion tasks (p-value = 0.072) and the subject 6 task 4 (little finger extension), for the extension task (p-value = 0.092).

The analysis of the intra-task variability, i.e., the similarity with which different subjects performed a specific task, showed good consistency across subjects for task 3 (ring finger flexion) and 5 (thumb adduction) of the flexion tasks, with a coefficient of similarity higher than 0.75. For the extensor, tasks 2 (index extension), 3 (middle extension), and 6 (thumb abduction) verified the same condition.

**Figure 3:**
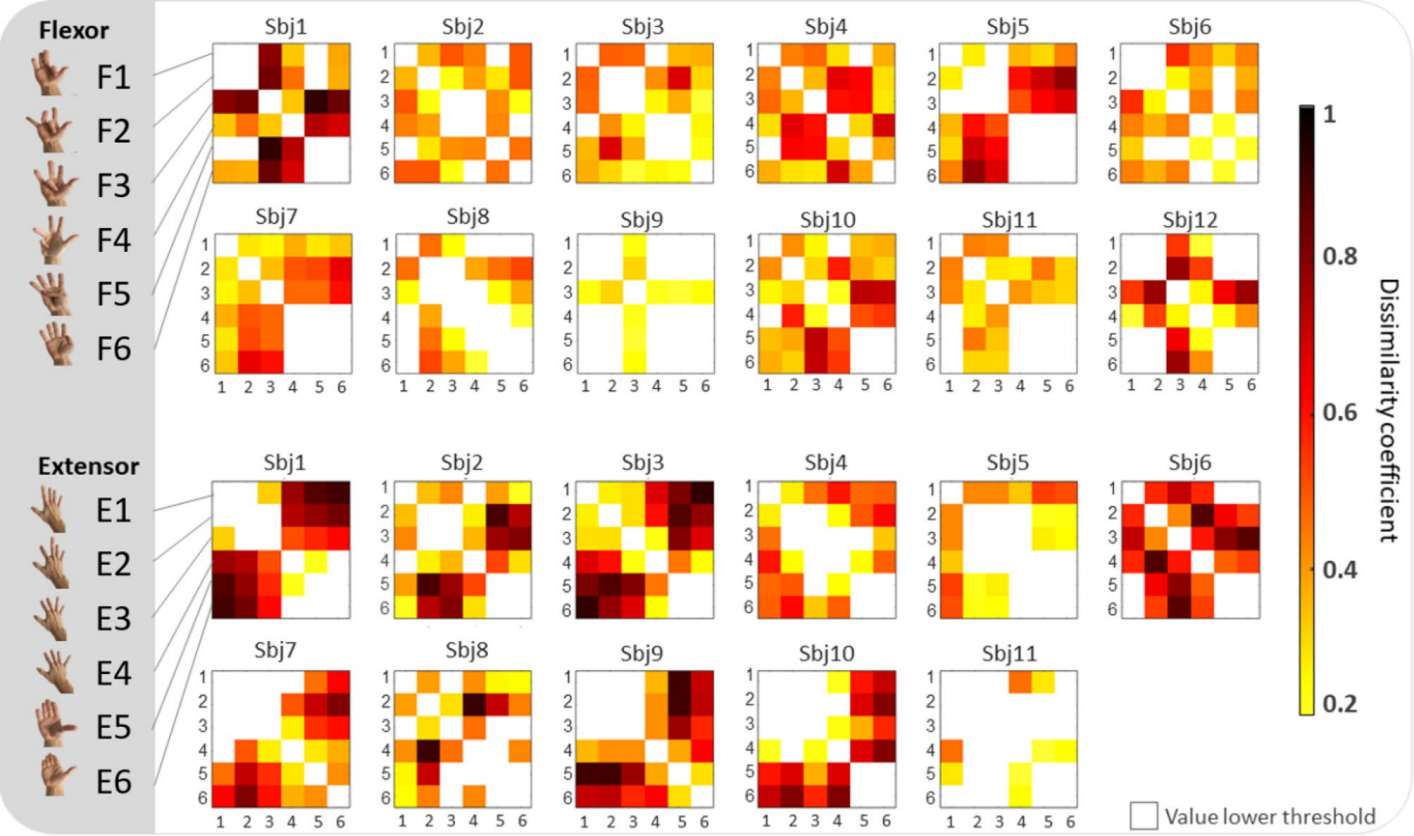
Intra-subject variability analysis for the flexion (F) and extension (E) tasks. The dissimilarity coefficient between pairs of gestures is shown in every subject, for the flexion (upper panel) and the extension (lower panel) sessions, separately. Values below 0.2 are not represented. Figure created with MatLab2020a and Microsoft PowerPoint 2016.

Figure 3 shows the results of the intra-subject variability analysis. The dissimilarity coefficient between pairs of gestures is shown for every subject, for the flexion and the extension sessions, separately. For the intra-subject dissimilarity analysis, a minimum threshold of 0.2 was selected to identify movement which differs robustly. For flexors, subject 4 shows almost perfect distinctiveness between different tasks (with a dissimilarity index exceeding 0.2 for all combinations of task pairs, except pair 1–5). For extensor, subject 3 shows almost perfect distinctiveness between the different tasks (with dissimilarity index exceeding 0.2 for all different task pairs, except pair 5–6).

Overall, for both extension and flexion sessions, the subset of tasks 4, 5, 6 is distinguishable from subset 1, 2, 3, while the dissimilarity coefficient between tasks 5 and 6 (both including the thumb) is below the established threshold for almost all subjects.

### Classification performance

**Figure 4:**
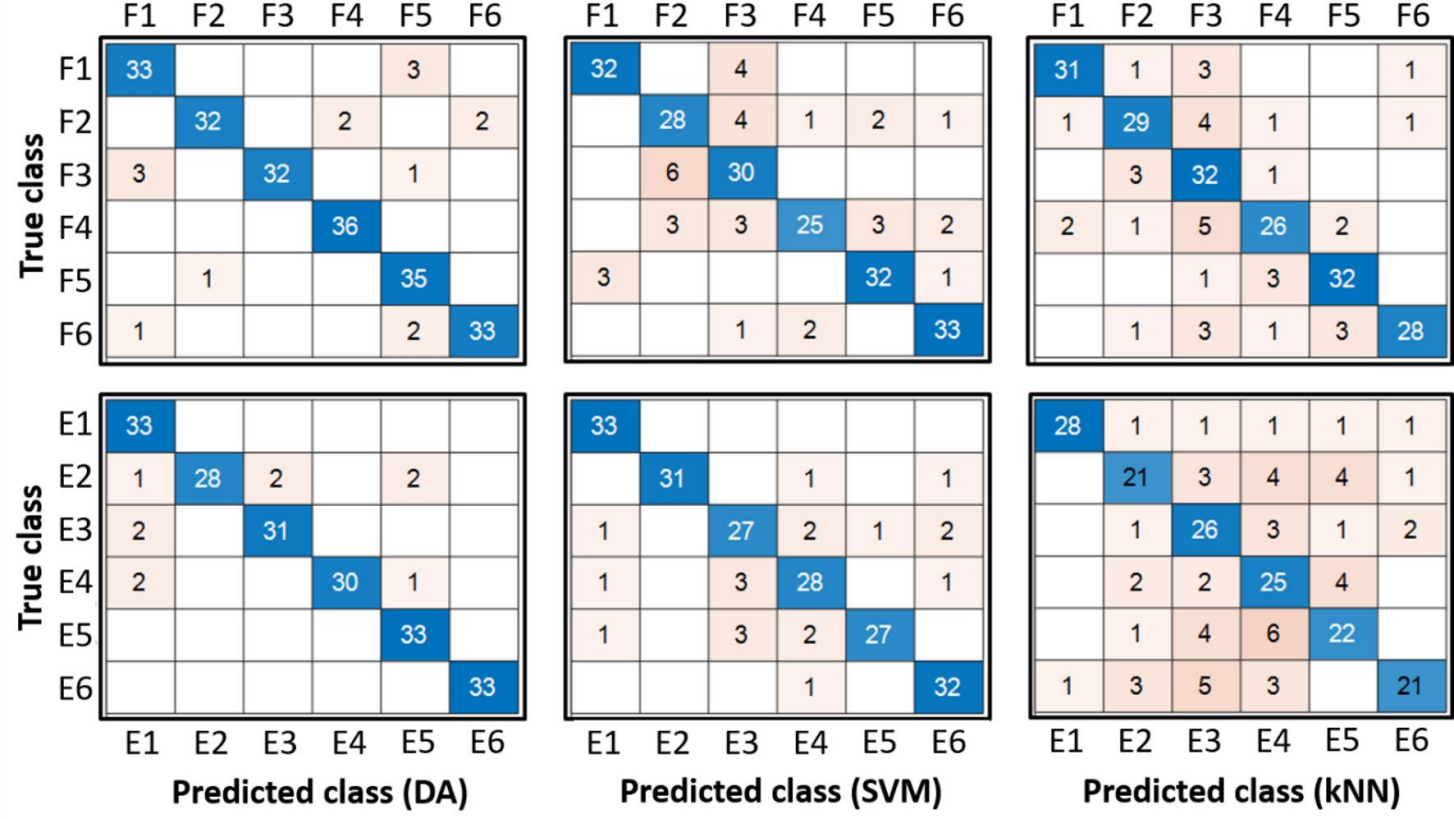
Confusion matrices were obtained in the multi-class classification problem for the flexion (F) and extension (E) tasks. Flexion (upper panel) and extension (lower panel) gestures were analyzed separately. From left to right, DA, SVM, and kNN results are shown. The average accuracy value for each of them is as follows: Flexion: 93% for DA, 83% for SVM, 82% for kNN; Extension: 95% for DA, 90% for SVM, 72% for kNN. Figure created with MatLab2020a and Microsoft PowerPoint 2016.

The confusion matrices obtained during the multi-class classification problems, i.e., for extension and flexion sessions, separately, are shown in Fig. 4. Performances were evaluated on the test set (fully independent from the training set), as described in Materials and Methods.

Figure 4 shows the correct classifications on the main diagonal of the confusion matrices and the misclassifications outside. For the flexion session, we obtained an average accuracy value of 93% for DA, 83% for SVM, and 82% for kNN. We also computed the average precision for every classifier, ranging from 85.4% to 100% for DA, 71.4% to 91.4% for the SVM, and 66.7% to 93.3% for kNN, depending on the specific gesture. Similarly, the average recall ranges from 88.9% to 100% for DA, 69.4% to 91.7% for SVM, and 72.2% to 88.9% for kNN, depending on the specific gesture.

For the extension session, we obtained an average accuracy value of 95% for DA, 90% for SVM, and 72% for kNN. We also computed the average precision for every classifier, ranging from 86.8% to 100% for DA, 81.8% to 100% for the SVM, and 59.5% to 96.6% for kNN, depending on the specific gesture. Similarly, the average recall ranges from 84.8% to 100% for DA, 81.8% to 100% for SVM, and 63.6% to 84.8% for kNN, depending on the specific gesture. Therefore, the classification of the five different fingers reaches accuracies above 90%, comparable with the most recent literature^19–22^. In particular, in one of these works, the authors acquired sEMG using an 8-channels MYO band and classified five finger movements and rest periods using several classifiers, including kNN and SVM, reaching a maximal accuracy of 95.8%^22^. They also reported accuracies in the range 66–92% from previous related literature. We found relatively low SNR values as in Shaabana et al. 2019^23^. Nevertheless, we were able to find satisfactory classification performance. Accuracy reaches satisfactory levels, but precision and recall were also found above 90% for most fingers with this type of classifier. This also is in line with Shaabana et al., where precision and recall were found in the range 64–95%.

We have proved the feasibility of a fatigue detection study using the same inkjet-printed technology. Interestingly, we could classify with good levels of accuracy the same gestures across different subjects without having assessed their MVC. This could be useful in the case of patients that cannot exert MVC or in case real-time acquisition is performed without preliminary calibration on MVC.

Compared with previous literature^24–28^, in this research work, we introduced a detailed investigation of the quality of the sEMG signals we acquired via inkjet-printing. In the most recent studies^30–34^, however, more attention has been dedicated to evaluating the repeatability of a sEMG pattern during the same task. In Pizzolato et al.^34^, the signal amplitude was analyzed across days and compared through six different sEMG setups. This analysis could favour the design of personalized inkjet-printed matrices for specific motor control applications. Furthermore, the combination of this technology with the recent advancements in Finite Element Models (FEM) simulations^33–35^ may permit obtaining unified algorithms for the automatic design of optimal distributed electrodes’ matrix with respect to the peculiar characteristics of each patient.

## Outlook

This paper exploited a low-cost sensor printing system based on silver nanoparticle (AgNP) inks^36–38^ and a commercial inkjet printer to obtain an 8-channel surface EMG matrix. In a previous study, we investigated the reliability of inkjet-printed electrodes for a single EMG sensor, compared to Ag/AgCl standard electrodes, and implemented a robust fatigue study with six subjects performing repetitions of a weight-lifting task^14^. Here, we extended our preliminary study and designed a matrix of inkjet-printed sEMG sensors. Then, we investigated and discussed in detail the quality of the sEMG signals acquired from 12 subjects during six different flexion and extension finger movements. We also assessed the robustness of the sEMG patterns across repetitions and subjects and performed gesture recognition classification (flexion and extension, separately).

Our results showed the reliability of our sEMG setup, as we could obtain high classification accuracy (93–95%), precision, and recall values, both in the flexion and extension finger movements. Moreover, we found a significant similarity level (measured by Spearman’s correlation-based similarity index) across repetitions of the same movement in every subject and dissimilarity (measured by Spearman’s correlation-based dissimilarity index) large enough to discriminate between different movements in every subject. Therefore, we have proved that our inkjet-printed sEMG matrix provides signal quality comparable to current research-purpose sEMG devices and allows effective gesture recognition.

Beyond that, its ultra-short design-to-prototype time (“print and play”), its scalability towards a higher number of channels, and its customization possibilities (in terms of geometry and size of the electrodes) make it suitable for any clinical settings, avoiding complicated fabrication procedures, regardless of the availability of expensive equipment materials and without the need of advanced technical expertise.

As proved by previous works, many machine interfaces are available in presence of a good signal transduction and appropriate and accurate classification method. These include prosthetics^39,40^, exosuits^41^, haptic feedback^11^, vocal synthesis^10^, the control of robots and drones, virtual and augmented reality interaction^10^. Conversely, the EMG signal transduction remains a critical part of the system, particularly for applications requiring customized electrodes geometries adapting to the specific patient needs.

Our work contributes to the research efforts focused on developing tools to evolve from a laboratory setting to wearable applications, i.e., towards scalable solutions to assist people, both patients, and caregivers, to improve or recovery from neuromotor diseases or injuries. Further customization could be investigated by using FEM simulations to personalize the sEMG acquisition setup to make it suitable for different purposes, depending on the needs, e.g., less obtrusive for continuous monitoring during daily living or a denser setup for very fine clinical tests during motor training.

## Methods

### Design and fabrication

The design and fabrication of the devices were performed following the method and protocols previously defined and optimized for the production of inkjet-printed impedance-based sensors^36–38^ and SEMG electrodes^14^. The only variation was the production of the insulating layer with patterned adhesive sheets, detailed below.

**Figure 5:**
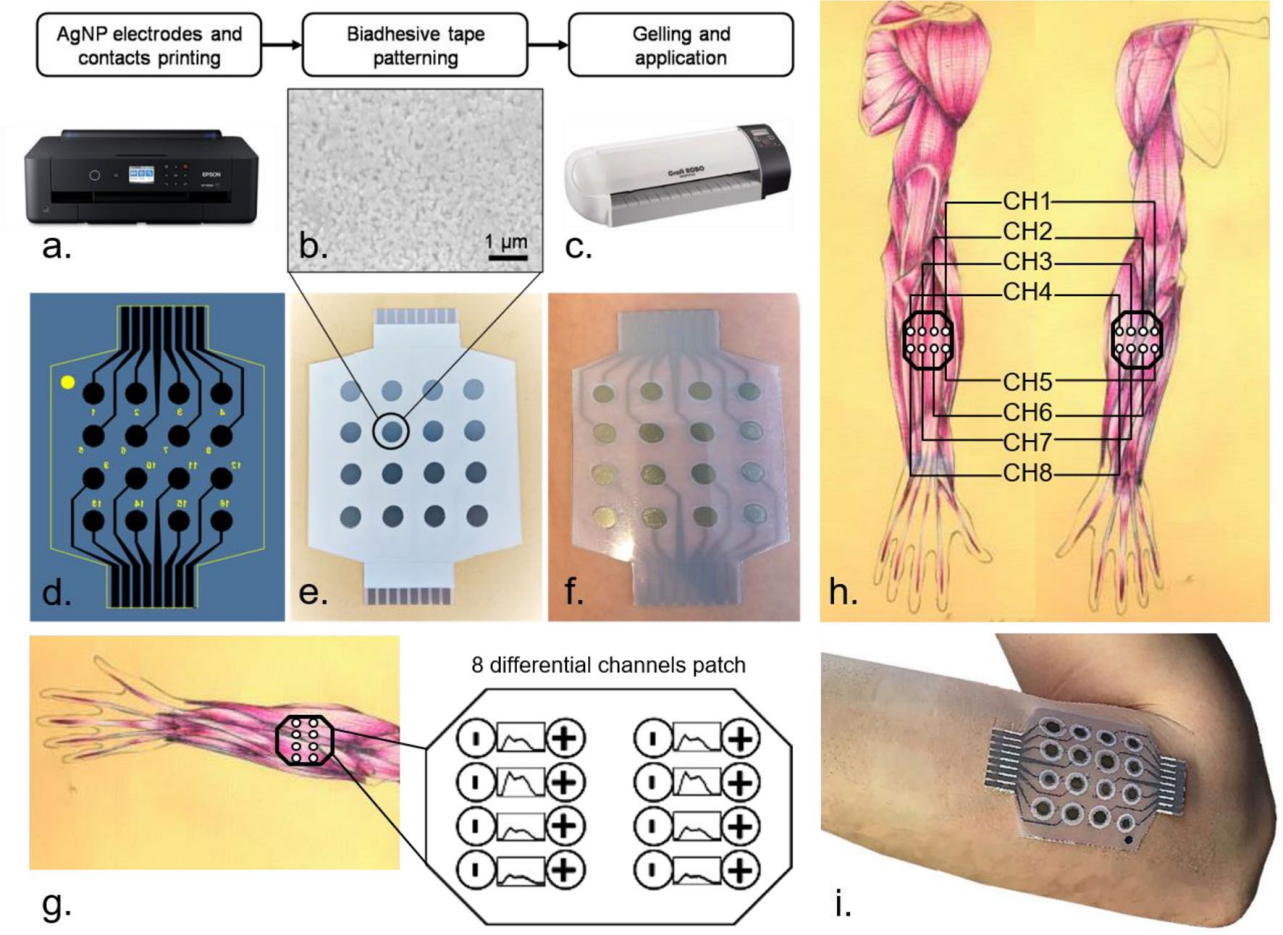
Graphical representation of the fabrication process of the electrodes’ matrix and their positioning on the forearm. (**a**) The Epson Stylus 1500 W consumer printer used for the printed sensors, loaded with AgNP and standard black ink. (**b**) SEM image of the printed AgNPs. (**c**) CraftROBO C330 digital blade cutter for the patterning of the biadhesive sheet. (**d**) AutoCAD matrix design with cutting lines and mirrored text to identify the electrodes through the transparent PET film after its application on the skin. (**e**) Printed electrodes’ matrix with the biadhesive passivating layer sticked on top. (**f**) Matrix of electrodes gelled and ready to be positioned on the participant forearm. (**g**) Example of the matrix positioning for extension tasks and of the readout of the signals between the electrodes, forming the 8 channels (arm original drawings courtesy of Maria Catalina Li Puma). (**h**) Readout channels positions for extension (right) and flexion (left), (arm original drawings courtesy of Maria Catalina Li Puma). (**i**) Matrix positioned on the forearm of one of the subjects. Figure created with Microsoft PowerPoint 2016.

All the device layouts were designed in Autodesk AutoCAD 2018 with black representing the silver nanoparticles ink areas and using yellow (cartridge loaded with standard Epson black ink) for text and cut lines. The layouts were printed directly from the software by a consumer printer (Epson Stylus 1500W, Fig. 5a) opportunely loaded with Mitsubishi Paper Mills Silver nanoink on pre-coated PET film from the same company. The film coating is optimized for the silver nanoparticles (AgNP) ink, allowing it to rapidly sinter at room temperature in a process called chemical sintering. In this process, the silver nanoparticles (average diameter of 150 nm, Fig. 5b), which are covered by a proprietary thin organic layer, accumulate on the coating surface, which acts like a membrane and filters the nanoparticles. At this point, the solvent recalls a reagent from inside the coating by osmosis. This reagent removes the organic layer from the nanoparticles leaving their conductive surface exposed and in tight contact with the other nanoparticles.

For the production of the passivation layer, biadhesive plastic sheet (Tosingraf, Vicenza, Italy) were patterned with a CraftROBO C330 (Fig. 5c) with a design produced in the respective ROBOmaster software, coherently with the one developed in AutoCAD (Fig. 5d) for the electrodes and the contacts. The passivation adhesive layer had the dual goal of leaving exposed only the electrodes, passivating the respective contacts, and to allow the device to adhere perfectly to the skin of the participants.

Thanks to previously printed registration marks, the biadhesive layer was aligned to the AgNP printed matrix and stuck in place (Fig. 5e). Right before the matrix application on the participants’ skin, the electrodes were covered with 2 ul of gel and the biadhesive liner was peeled off (Fig. 5f). Then the matrix was applied on the top or bottom part of the forearm for extension or flexion movement signal recording (Fig. 5g,h,i).

### Electrode geometrical characteristic tests

The geometrical characteristics of the electrodes were investigated to define the inter-electrodes distance and the electrode’s diameter resulting in the higher sEMG signal quality. With this purpose, matrices of 3 electrodes, respectively positive ( +), ground (GND), and negative (-) were printed with electrode diameters of 5 mm and 7.5 mm, and inter-electrodes distances (center-to-center) of 10 mm, 20 mm and 50 mm. All the matrices were applied on the forearm of the same participant and tested while performing the repeated lifting of a 5 kg weight for 3 s. Adequate resting periods were performed to ensure the avoidance of any fatigue mechanism to influence the acquisitions.

### Experimental protocol and data acquisition

In order to test the reliability of the newly fabricated electrodes, we recruited 12 healthy participants (28.42 ± 4.36 years old). During acquisitions, a matrix of sensors with 8 bipolar channels was arranged on their forearm. The experimental protocol included two sessions: in session 1, the matrix was placed on the anterior side of the forearm; in session 2, it was placed on the posterior side of the forearm. The specific location for attaching the inkjet-printed matrix varied according to anatomical characteristics and it was identified by palpation of flexor/extensor muscles during index finger flexion/extension. A landmark for each session and each subject could then be defined. The matrix’s channels 1 and 5 were positioned on the anatomical landmark. A ground standard electrode was placed at the wrist of the non-dominant hand.

**Figure 6:**
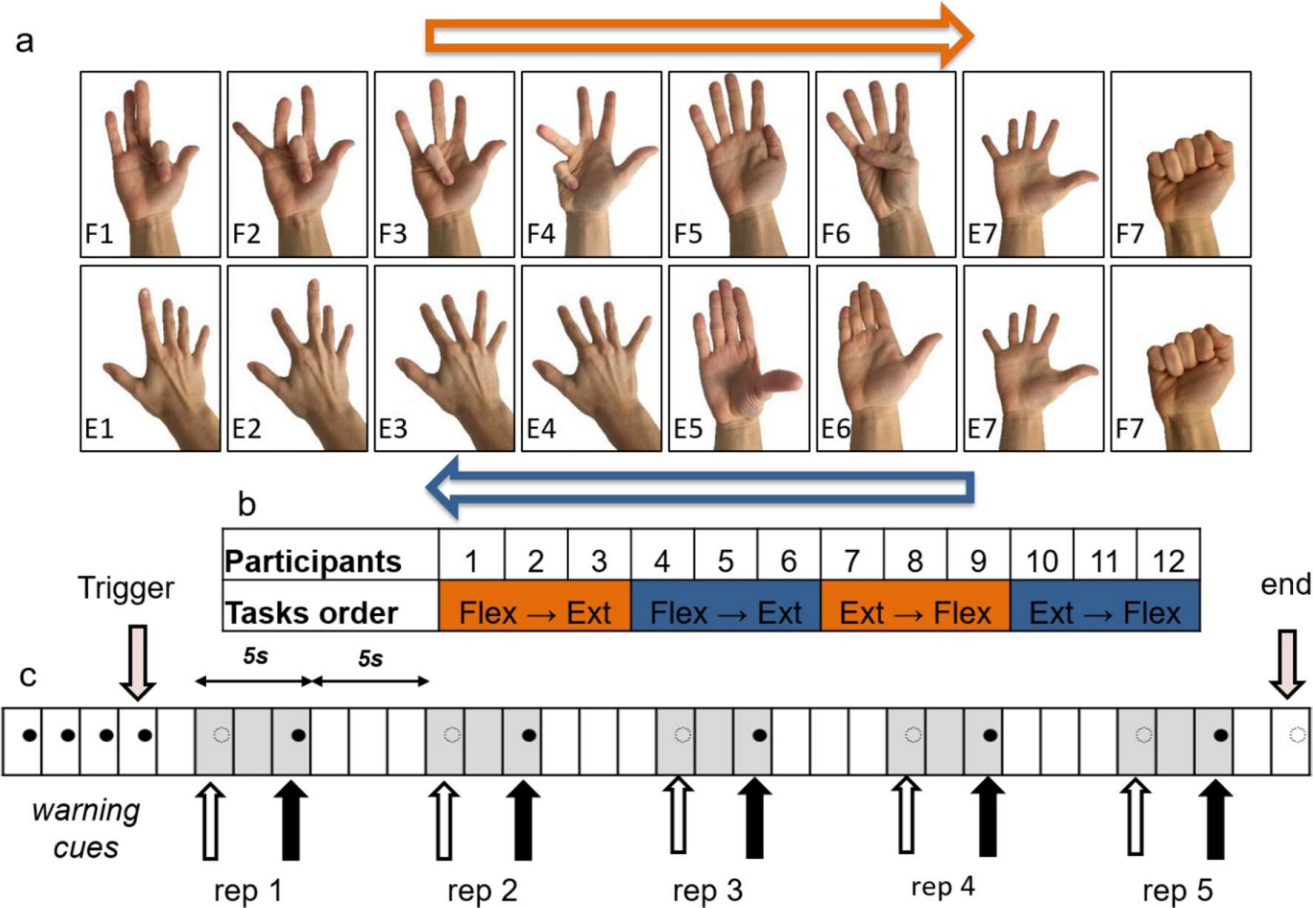
16 gestures performed by the 12 participants, randomization of their performance and structure of each acquisition of each gesture. The last two gestures in each row are the same and have been used as control movements. (**a**) Visual description of the gestures and of their execution order (depending on the subject group). To each gesture, it has been assigned a univocal code composed by a letter (F for flexion and E for Extension) and a progressive number. (**b**) Table identifying the muscle group and task execution order for each participant. (**c**) schematic of the acquisition performance for each gesture. Figure created with Microsoft PowerPoint 2016.

Participants were introduced to the experimental task with detailed instructions and a training simulation. The experimental protocol involved the execution of 12 individual finger movements and two hand movements. All of them were selected in the DB-1 Ninapro database^42^. Every participant performed two sessions: in one of them, the sEMG matrix was placed corresponding to of the extensor muscles and participants performed 6 individual finger extensions and the 2 hand movements (common to both sessions and used as control movements); in the other protocol, they performed 6 individual finger flexions and the 2 hand movements. All, but the thumb, were involved once in the movements. In contrast, two movements involved the use of the thumb. The resting position was held by keeping the hand open (palm up for flexion, and palm down for extension), with the wrist held over a rigid object at the height of the elbow (90 degrees arm flexion). The session order was randomized across participants. Within a session, the same order of movements was performed, with 5 repetitions per movement. At each repetition, an audio file instructed the participant when to start the movement and, when to stop it. At the very beginning, four short acoustic cues were produced, followed by a short silent pause. Then, a different warning cue allowed the participant to start the movement. Finally, another acoustic cue required him/her to go back to the resting position. Each extension or flexion position was kept for 5 s. An interval of 5 s was interleaved between two consecutive repetitions, in order to make the participant relax. Additionally, 1 min of resting was scheduled between two different movements to avoid muscle fatigue. At the end of each cycle, the matrix was removed and replaced with a new one. Figure 6a displays the experimental protocol and all required movements.

This study was conducted in accordance with the Declaration of Helsinki and approved by the Ethics Committee of Santa Lucia Foundation IRCCS, Italy (Prot. CE/PROG.804). Informed consent was obtained from all the participants and all the experiments were performed in accordance with the relevant guidelines and regulations.

### sEMG acquisition setup

The inkjet-printed sEMG matrix was positioned corresponding to either the flexor, or the extensor, muscles of the participants’ forearm. The eight bipolar channels of the matrix were aligned over the Flexor carpi radialis, the Palmaris longus, the Flexor digitorium superficialis and also a part of the Flexor carpi ulnaris muscles (Fig. 5h). The first and third proximal rows of the matrix were connected to the positive poles of each channel, while the second and fourth rows were connected to the negative ones. The signals were measured between the first two and last two rows (Fig. 5g).

Each matrix was connected to the acquisition board by a couple of shielded flat wires which had been inkjet printed with the same protocols used for the matrix fabrication and then insulated with biadhesive tape and PET. The acquisition board, allowed the tripolar channels of the amplifiers to be connected to the matrix, and collected all the amplifiers channels ground to a singular electrode placed on the wrist of the participant, over the bone, to avoid any interference. Finally, the acquisition board was equipped with a switch connected to the amplifiers triggers to synchronize the acquisitions.

The amplifiers used were two Biopac MP35 (BIOPAC Systems, Inc., Goleta, CA, USA) and all the SEMG signals were acquired with sampling frequency of Fs = 2 kHz and resolution of 12 bit.

### Pre-processing

All sEMG signals were acquired with a sampling frequency of FS = 2 kHz and filtered with a band-pass filter (Hamming window, order 4) in the frequency band 10—380 Hz. Power-line interference was removed with a notch filter (band-stop filter between 49 and 51 Hz using a fourth-order Butterworth filter). Systematic and random reaction times variations caused some misalignment between the audio stimulus and the actual movement performed by the participants, so we chose to extract EMG segments corresponding to the periods of time where the real EMG contraction was exerted: therefore, five 3 s-long segments were obtained from the task session. For the sake of visualization, rectification and smoothing were applied to the collected data using a moving average filter with a fixed 100 ms window.

### Feature extraction

Well-established time- and frequency-domain features were extracted from the pre-processed dataset^43,44^. The feature matrix was composed of feature vectors extracted from bioelectric signals recorded during the execution of movement of the individual fingers using 1 s non-overlapping specific time windows, so from each 3 s repetition of the finger movement we collected 3 feature vectors. Each feature vector was composed of 88 observations obtained extracting 11 features for each channel. The feature matrix was used in order to assess the possibility of classifying the movement of individual fingers both for flexion and extension.

Specifically, we computed the mean amplitude value (*MAV,* Eq. (1)); the maximum value (*MAX* = *max{x}*); the minimum value (*MIN* = *min{x}*); the range (*RANGE* = *MAX–MIN*); the root mean square (*RMS*, Eq. (2)); the variance (*VAR*, Eq. (3)); the standard deviation (*STD,* Eq. (4)); the zero crossing (*ZC*, Eq. (5)) that counts the number of times the signal crosses zero or changes sign; the waveform length (*WFL*, Eq. (6)) that provides information on the waveform complexity in each segment; the mean frequency (*MNF*, Eq. (7)) that is an average frequency value computed as a sum of product of the EMG power spectrum and frequency, divided by a total sum of spectrum intensity; and finally the median frequency (*MDF*, Eq. (8)) that is a frequency value at which the EMG power spectrum is divided into two regions with an equal integrated power.

1 $$MAV = \frac{1}{N}\mathop \sum \limits_{i = 1}^{N} \left| {x_{i} } \right|$$

2 $$RMS = \sqrt {\frac{1}{N}\mathop \sum \limits_{i = 1}^{N} x_{i}^{2} }$$

3 $$VAR = \frac{1}{N - 1}\mathop \sum \limits_{i = 1}^{N} \left| {x_{i} - \mu } \right|^{2}$$

4 $$STD = \sqrt {\frac{1}{N - 1}\mathop \sum \limits_{i = 1}^{N} \left| {x_{i} - \mu } \right|^{2} }$$

5 $$ZC\left( z \right) = \mathop \sum \limits_{i = 1}^{N - 1} s \left( {x_{i} , x_{i - 1} } \right)\;{\text{with}}\;s\left( {z, y} \right) = \left[ {\begin{array}{*{20}c} 1 & { if \left( {z \cdot y} \right) < 0} \\ 0 & { otherwise} \\ \end{array} } \right]$$

6 $$WFL = \mathop \sum \limits_{i = 0}^{N} \left| {x_{i} - x_{i - 1} } \right|$$

7 $$MNF = \frac{{\mathop \sum \nolimits_{i = 1}^{N} f_{i} \cdot PSD_{i} }}{{\mathop \sum \nolimits_{i = 1}^{N} PSD_{i} }}$$

8 $$MDF : \mathop \sum \limits_{i = 1}^{MDF} PSD_{i} = \mathop \sum \limits_{i = MDF}^{N} PSD_{i} = 1/2\mathop \sum \limits_{i = 1}^{N} PSD_{i}$$

*PSD*_*i*_ is the EMG power spectral density at a frequency bin *i* and *N* is the length of the frequency bin. Then, frequency analysis provides important information on fatigue assessment and on the contributions of both slow muscle fibers specialized in maintaining a position and fast muscle fibers particularly active during the execution of a motor task^45^. To compute *MNF* and *MDF*, the power spectral density (*PSD*) of the signal during task and rest segments, separately, was estimated via Welch’s periodogram. A 1 s Hamming sliding window (overlap rate of 50%) was used to obtain the power spectral decomposition with a frequency resolution of 0,9766 Hz in the frequency range 20–300 Hz. Additionally, we computed the signal-to-noise ratio (*SNR* = 10 log_10_ [(*P*_*signal*_*-P*_*noise*_)/*P*_*noise*_])^46^.

### Visual inspection of the dataset

First, we visually inspected the raw data and the bubble plots to qualitatively evaluate any clear differences among the different movements and different subjects. Time and frequency domain inspections were carried out, both in the rest and task periods.

Second, we evaluated the variability of our dataset through the statistical description of every feature extracted from the raw data (not reported here for space constraints). Then, we investigated the intra-subject and inter-subject variability in the different movements, through feature representation of the sEMG signal samples. Finally, we classified the 6 individual finger movements using three different classifiers.

### Intra-subject and inter-subject variability

In order to assess the robustness of our inkjet-based sEMG measurements across the same participant, we performed a correlation analysis across the repetitions of the same movement in the same participant. As already mentioned, the RMS feature is frequently used as signal activity metrics in sEMG, thus we used it to evaluate the repeatability of a movement across repetitions and participants. Also, we limited this analysis to the 6 individual finger movements of each session, thus excluding the hand movements. The extension and flexion sessions were analysed separately.

From each repetition, as explained in the features extraction section, we extracted three 8-dimensional RMS values (i.e., three 1 s -windows, 8 sEMG channels), and from each movement we obtained fifteen 8-dimensional RMS values. The latter were normalized over their maximum value across repetitions and channels.

Then, for each participant *i* = 1, …, 12 and each kind of movement *m* = 1,…, 6, we defined a single normalized 8-dimensional RMS value as a pattern and we represented it by *x*_*i,m*_*(k)* = *[x*_*i,m,k*_(1),* x*_*i,m,k*_(2), …, *x*_*i,m,k*_(8)], with *k* = *1, .., 15* the observation index (corresponding to a window in a repetition of movement *m* of participant *i*).

To evaluate the similarity between two different movement repetitions, we calculated the Spearman’s correlation coefficient between *x*_*i,m*_*(k)* and *x*_*i,m*_*(q)*, with *k ≠ q*.

$$r_{i,m} \left( {k,q} \right) = 1 - \frac{{6\sum d_{i,m} \left( {k,q} \right)}}{{n\left( {n^{2} - 1} \right)}}\;{\text{with}}\;\begin{array}{*{20}l} {n = n{\text{umber of observations}}} \hfill \\ {d = {\text{difference between the ranks of each observation}}} \hfill \\ \end{array}$$

We then aggregated the similarity values by averaging them across the repetitions, leading to 72 *r*_*i,m*_ values (one for each different movement and each participant). To assess the significance of each Spearman correlation coefficient, a statistical test was used. Specifically, t-tests were applied to the average similarity coefficients across repetitions to evaluate if for each subject there is a significant repeatability for a specific task.

Similarly, we evaluated the dissimilarity between two different movements. To do this, for every participant *i* = 1, …, 12 and every kind of movement *m* = 1, 2, …, 6, we obtained a normalized 8-dimensional vector, averaging across observations. We represented it by *x*_*i*_*(m)* = [x_i,m_(1), x_i,m_(2), …, x_i,m_(8)].

The dissimilarity coefficient was calculated using the metrics *1-r*_*i*_*(m,n)* , between *x*_*i*_*(n)* and *x*_*i*_*(m)*, with *m ≠ n* and *m,n* = 1, 2, …, 6, so we obtained a dissimilarity value for every pair of movement *(m,n)* with *m ≠ n* and for each participant *i*.

### Classification

From each repetition, movement, and participant, as explained in the feature extraction section, we extracted three 11-dimensional samples (i.e., three windows, 11 features). Thus, for each movement, we collected 180 11-dimensional samples (15 samples × 12 participants), and this formed the feature matrix used as the classifier’s input.

Then, we performed a six-class classification to assess the possibility to distinguish the movement of individual fingers from the inkjet-printed sEMG measurements. Flexion and extension sessions were considered as separate classification problems.

To evaluate the classification performance in a reliable way, we split the entire dataset into a training and a test set (independent from the training one). The training set included repetitions 1, 2, 4, and 5 of each movement from all participants. The test set contained the third repetition. This choice can be justified by considering that repetition 3 was the one where the participants might not be fully used to the task, yet, and at the same time they might not have already experienced fatigue from multiple repetitions of the same task. Incidentally, previous literature adopted a similar choice^27^.

Three different classifiers were selected to solve the two classification problems: a support vector machine (SVM), a k-nearest neighbour (k-NN) and a discriminant analysis (DA). We used Matlab to implement all of them. The hyperparameters of each model were optimized via a tenfold cross-validation on the training set (by the inherent Matlab’s optimization tool in the Classification Learner App, available in Matlab 2019b). Particularly, for the SVM, the best kernel type and its hyperparameters were optimized by choosing the combination of values providing the best accuracy in the tenfold CV. For the k-NN, the k had to be tuned; and, for DA, linear or quadratic fit were tested.

The classification performance was computed in terms of averaged accuracy Eq. (9) precision Eq. (10), recall Eq. (11) and F1 score Eq. (12). In a multi-class classification problem, they are defined as follows^47^:

9 $$Accuracy = 1/C\mathop \sum \limits_{i = 1}^{C} \frac{{tp_{i} + tn_{i} }}{{tp_{i} + fn_{i} + fp_{i} + tn_{i} }}$$

10 $$Precision = 1/C\mathop \sum \limits_{i = 1}^{C} \frac{{fp_{i} + fn_{i} }}{{tp_{i} + fn_{i} + fp_{i} + tn_{i} }}$$

11 $$Recall = 1/C\mathop \sum \limits_{i = 1}^{C} \frac{{tp_{i} }}{{tp_{i} + fn_{i} }}$$

12 $$macroF1 = \frac{{2* p_{M} *r_{M} }}{{p_{M} + r_{M} }}$$

with C is the number of classes, $tp_{i}$ $fp_{i}$ $tn_{i}$ $fn_{i}$ are the number of true positives, false positive, true negatives, and false negatives, respectively, in the each class $i = 1,2, \ldots , C$. Moreover, $p_{M}$ and $r_{M}$ represent the macro-averaged value for precision and recall.

## Supplementary Information

Supplementary Information.

## Data Availability

The datasets generated during and/or analysed during the current study are available from the corresponding author on reasonable request.
