# Supplementary material for: Inkjet-printed fully customizable and low-cost electrodes matrix for gesture recognition
Source: Sci Rep. 2021 Jul 22;11:14938. doi: 10.1038/s41598-021-94526-5 (PMC8298403; doi:10.1038/s41598-021-94526-5)
Supplement: Supplementary file 1 — Supplementary Information. [file 41598_2021_94526_MOESM1_ESM.docx]

**Supplementary figures**

**Inkjet-printed fully-customizable and low-cost electrodes matrix for gesture recognition**

Giulio Rosati^*,+^, Giulia Cisotto^+^, Daniele Sili, Luca Compagnucci,

Chiara De Giorgi, Enea Francesco Pavone, Alessandro Paccagnella, Viviana Betti


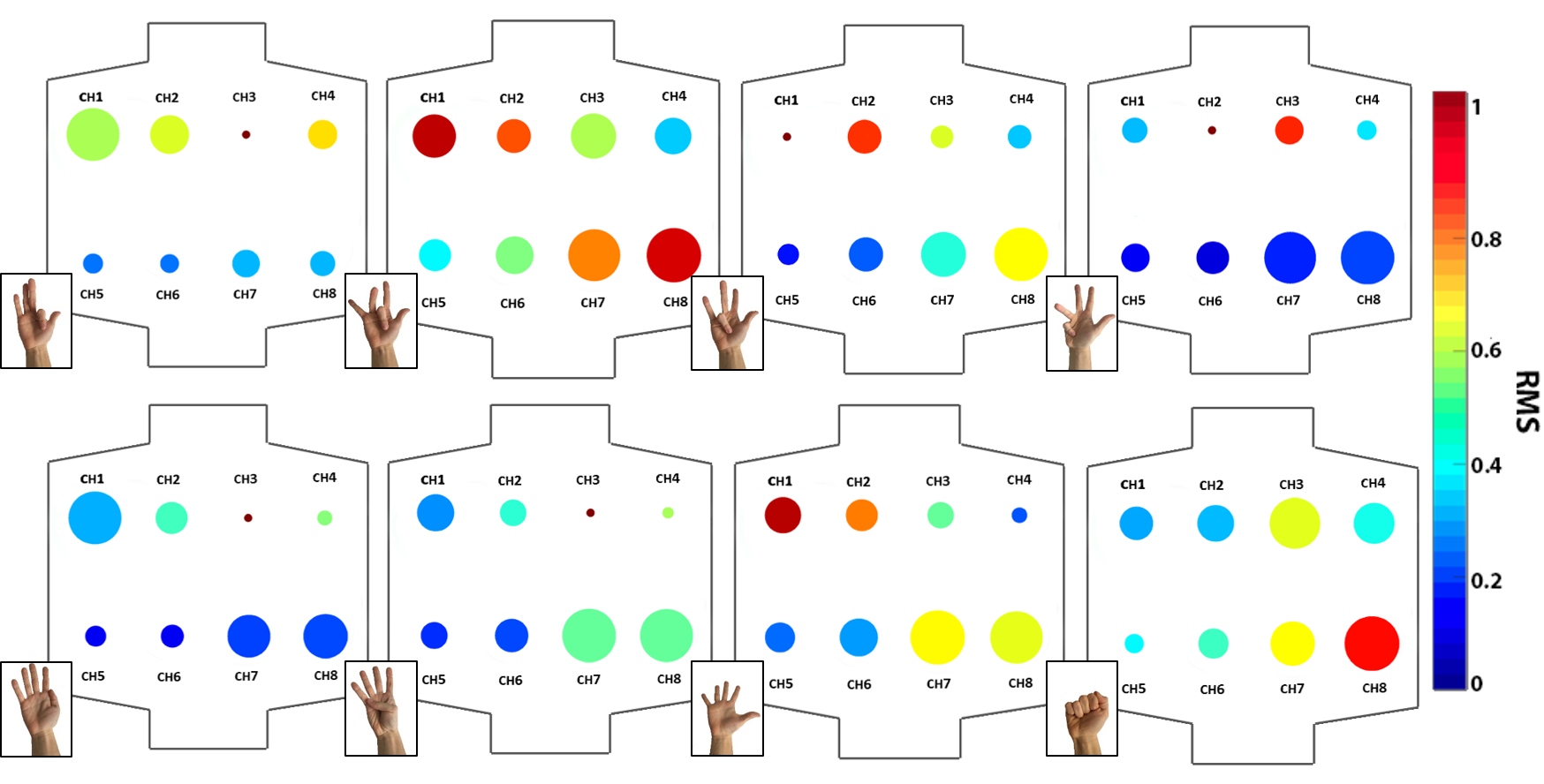


**Fig. S1.** Bubble plots of the 8 flexion tasks performed by subject 8. Figure created with Matlab 2020a and Microsoft PowerPoint 2016.


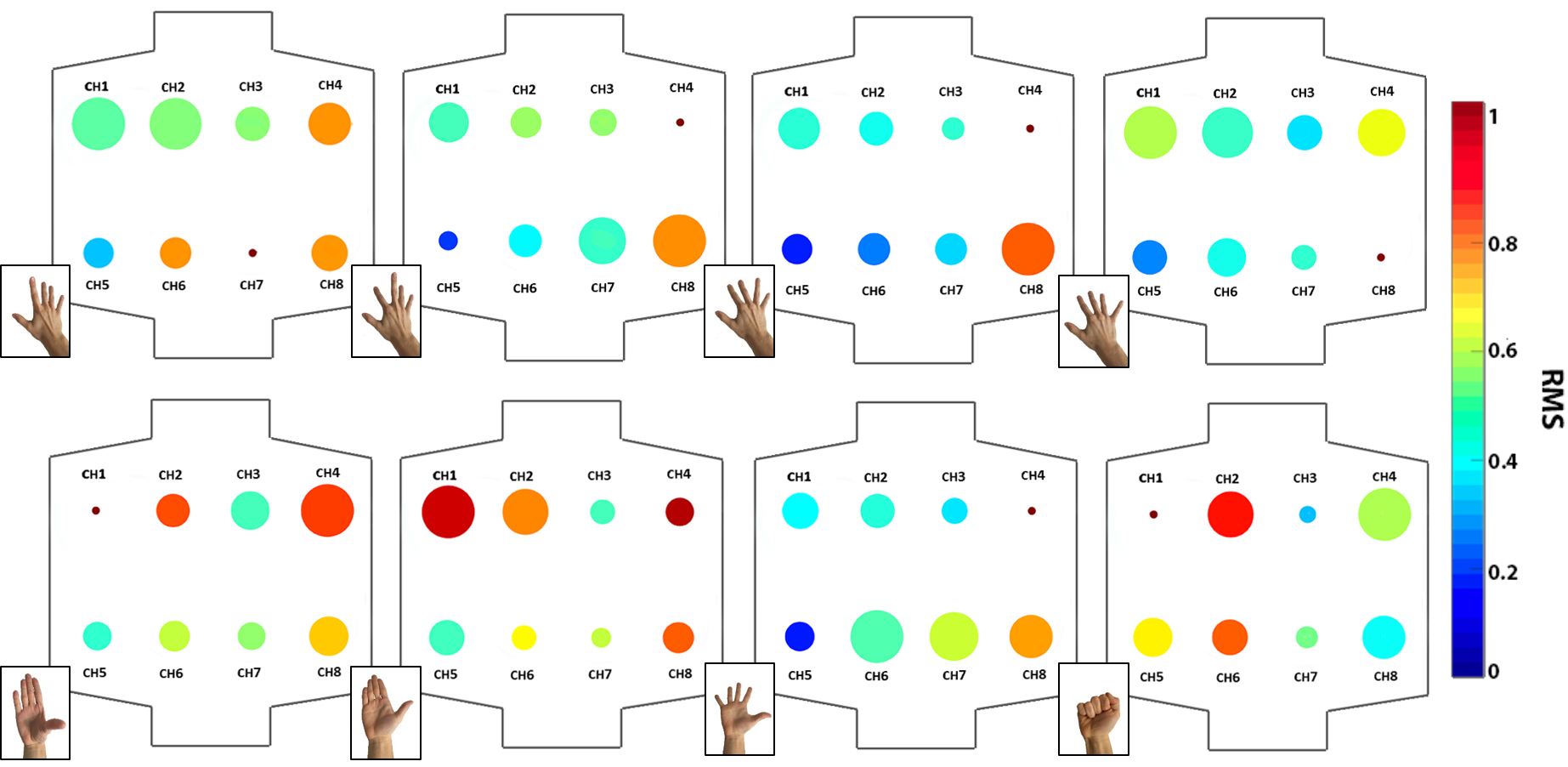


**Fig. S2.** Bubble plots of the 8 flexion tasks performed by subject 8. Figure created with Matlab 2020a and Microsoft PowerPoint 2016.


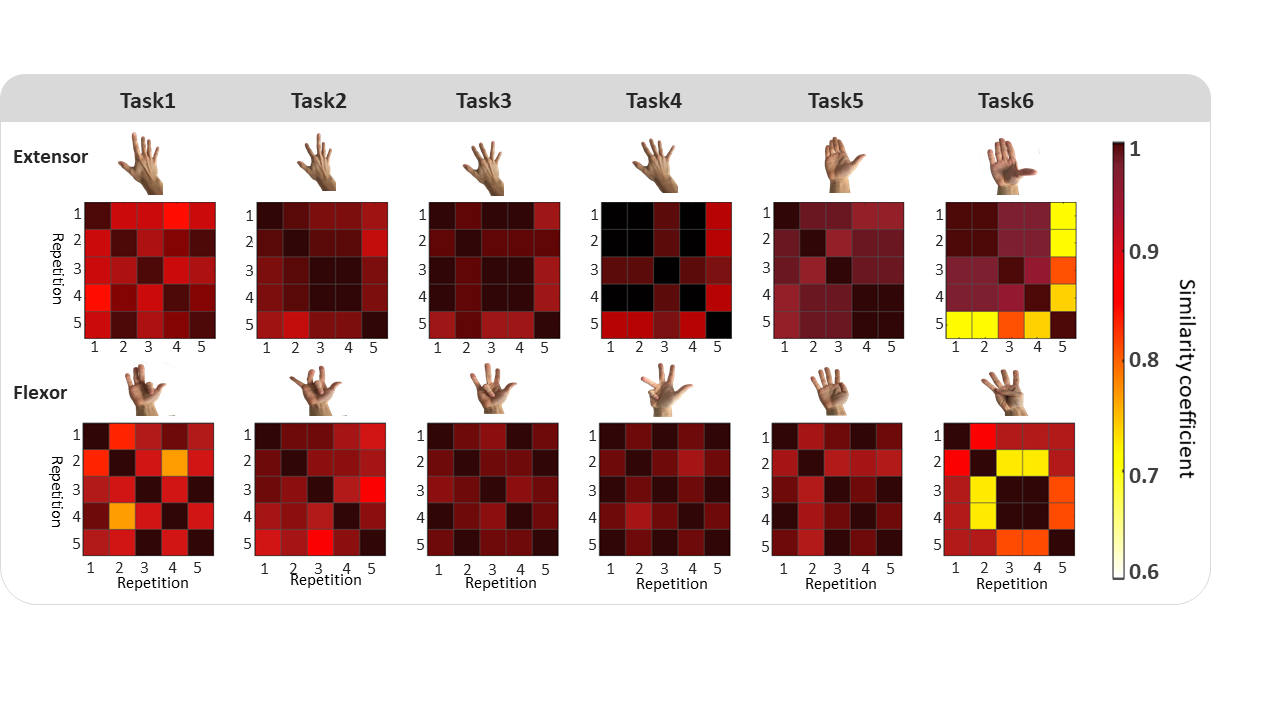


**Fig. S3.** Intra-subject similarity analysis. The similarity coefficient across each repetition  is shown for every gesture (task 1-6), for two different subjects, for the flexion (upper panel) the subject 8 is shown, while for the extension (lower panel) the subject 5 is shown. Note that the colorbar ranges are from 0.6 to 1. Figure created with Matlab 2020a and Microsoft PowerPoint 2016.
